# Unveiling immunity dynamics: Serological characteristics of antibodies against Japanese encephalitis virus in Guangdong, China

**DOI:** 10.1371/journal.pntd.0013629

**Published:** 2025-10-22

**Authors:** Yueling Chen, Runyu Yuan, Zixia Qian, Xinxin Li, Weizhao Lin, Can Xiong, Yingyin Deng, Chumin Liang, Huifang Lin, Limei Sun, Jianfeng He, Liang Chen, Ying Yang, Jiufeng Sun

**Affiliations:** 1 School of Public Health, Guangdong Pharmaceutical University, Guangzhou, Guangdong, China; 2 Guangdong Provincial Institute of Public Health, Guangdong Provincial Center for Disease Control and Prevention, Guangzhou, Guangdong, China; 3 Department of Public Health and Preventive Medicine, School of Medicine, Jinan University, Guangzhou, Guangdong, China; 4 School of Public Health, Southern Medical University, Guangzhou, Guangdong, China; 5 School of Public Health, Sun Yat-Sen University, Guangzhou, Guangdong, China; 6 Guangdong Workstation for Emerging Infectious Disease Control and Prevention, Guangdong Provincial Key Laboratory of Pathogen Detection for Emerging Infectious Disease Response, Guangdong Provincial Center for Disease Control and Prevention, Guangzhou, Guangdong, China; Public Health Agency of Canada, CANADA

## Abstract

**Background:**

Defining the immune dynamics of Japanese encephalitis (JE) in healthy individuals is crucial for assessing population susceptibility and evaluating the effectiveness of vaccinations.

**Methods:**

We conducted a cross-sectional serological survey of anti-JEV IgG antibodies and anti-JEV neutralizing antibodies (nAbs) in Guangzhou City, Zhanjiang City and Heyuan City of Guangdong Province, China.

**Results:**

A total of 691 participants were included from 2018–2022, among whom 50 were dengue IgG antibody positive and 641 were dengue IgG antibody negative. In the total population, the anti-JEV IgG antibody positivity rate detected by enzyme-linked immunosorbent assay (ELISA) was 51.37% (95% CI: 47.64–55.11%), and the neutralizing antibody positivity rate detected using the microneutralization test (MNT) was 73.22% (95% CI: 69.92–76.54%). Among the 641 dengue IgG antibody-negative subjects, the anti-JEV IgG antibody positivity rate by ELISA and the neutralizing antibody positivity rate by MNT were 48.05% (95% CI: 44.17–51.93%) and 72.07% (95% CI: 68.59–74.56%), respectively. Comparable geographical seroprevalences of either anti-JEV IgG or neutralizing antibody were observed in Guangzhou City, Heyuan City and Zhanjiang City, respectively (49.52% vs. 48.04% vs. 47.45%, 65.71% vs. 70.46% vs. 76.47%, respectively). Antibody positivity rates in all age groups exhibited a U-shaped curve, with the lowest rate occurring in the 7–18-year-old age group. With respect to the vaccine dose, the anti-JEV nAb positivity rate and geometric mean titer (GMT) detected by MNT were higher in those who received two doses of live attenuated vaccine than in those who received one dose or 0 doses (80.57% vs. 55.81% vs. 55.09% and 25.92 vs. 12.19 vs. 16.47, respectively). In the 641 dengue IgG antibody-negative subjects, moderate consistency between the MNT and ELISA results was observed (Kappa = 0.47, *r*_*s*_ = 0.76).

**Conclusion:**

The high seroprevalence in participants indicated a neglected transmission of JE, which highlights the importance of strengthening the surveillance of JEV in this area. The vaccination program against JEV is highly needed because of immune gaps in adults, e.g., boosters for adults aged 7–39 years.

## Author summary

Japanese encephalitis (JE) is an acute central nervous system disease caused by Japanese encephalitis virus (JEV) and is primarily prevalent in Southeast Asia and the Western Pacific. Vaccination is the most effective preventive measure. Although vaccination has significantly reduced the incidence of JE in children, the proportion of adult cases has been gradually increasing, highlighting the need for ongoing immune monitoring and strategic adjustments. In this study, we measured the levels of anti-JEV IgG antibodies and anti-JEV neutralizing antibodies (nAbs) in the serum of healthy participants in Guangdong Province, China, as well as their trends of change and correlations with vaccination. The seroprevalence distribution in Guangdong Province revealed a low seroprevalence among adolescents and a high seroprevalence among adults, indicating the potential for JEV transmission in the region. These findings indicate the need for enhanced JEV surveillance and adjustments to the vaccination program, particularly booster vaccinations for the 7–39-year-old age group. Overall, the findings of this study provide guidance for adjusting the Japanese encephalitis vaccination strategy.

## Introduction

Japanese encephalitis (JE), a zoonotic disease caused by Japanese encephalitis virus (JEV), is a major public health problem in Southeast Asia and the Western Pacific [1,2]. JEV, a single-stranded, positive-sense RNA virus, belongs to the genus *Flavivirus* in the family *Flaviviridae* [3]. JEV is transmitted by mosquitoes as a vector [4]. The most important vector of JEV in China is *Culex tritaeniorhynchus* [5]. Most JEV infections are asymptomatic. Only a minority of patients develop JE, with clinical manifestations such as acute onset, headache, fever, consciousness disorders and coma [6]. The fatality rate of patients with JE can reach 30%, and 30–50% of survivors may have permanent neurological or mental sequelae [7], leading to extremely serious health issues and social and economic burdens [8,9].

According to the World Health Organization (WHO), approximately 24 countries and 3 billion people worldwide are at risk of infection with JEV [10]. An estimated 67,900 cases occur per year, approximately half of which are in China [11]. All regions of China are JE endemic areas except for the Xinjiang Uygur Autonomous Region, Tibet Autonomous Region and Qinghai Province [12]. There is no effective treatment protocol for JE in the clinic. Vaccination against JE remains the best control strategy [13], which has been carried out in China since the mid-1970s and was updated in September 2008 [14]. The leading vaccines currently used in China are the JE live-attenuated vaccine (JEV-L), which was marketed in 1988, and the JE inactivated vaccine (JEV-I), which was marketed in 2008 [15]. The SA 14-14-2 live-attenuated vaccine has been included in the national immunization program with a schedule of primary immunization at 8 months and booster immunization at 2 years of age. According to a survey on routine immunization coverage among age-appropriate children in China, the coverage rates for one- and two-dose JEV-L vaccinations exceeded 99% in 2021 [16]. Vaccination has significantly reduced the incidence of JE in China, causing it to decrease significantly from 20.92 per 100,000 in 1971 to 0.0104 per 100,000 in 2022, and the number of deaths and mortality rates of patients have also shown downward trends [17]. As expected, there has been a significant decline in cases among children under 15 years of age [18,19].

Although live attenuated vaccines provide highly effective protection for several years after vaccination [20], the level of JEV-specific neutralizing antibodies (nAbs) decreases over time [21]. According to the WHO guidelines, protection against wild-type JEV infection is no longer possible if the nAb titer in human antisera falls below a protection threshold (1:10) [22,23]. Currently, JEV-L has been used in only a few Asian countries, but few studies have investigated its long-term immunogenicity. Interestingly, in India, a single dose of JEV-L provided adequate protection for at least 6 years, with vaccine efficacy declining from 91% (95% CI: 73.0–97.0) in the first year of vaccination to 71% (95% CI: 21.0–90.0) at six years after vaccination [24]. In Nepal, a single dose of JEV-L can provide protective neutralizing antibodies that persist for 1 year in 98.5%, 4 years in 89.9%, and 5 years in 63.8% of individuals [25]. These studies revealed geographical differences for single-dose vaccination. In China, Ran Wang et al. conducted a cross-sectional study on 961 residents aged 19–20 years in Beijing and reported that more than half of adults still had serum protection for nearly 20 years after receiving a live attenuated vaccine [26].

Guangdong Province, located on the southern coast of China, belongs to the East Asian monsoon region and is a hyperendemic area for JEV. Following the first epidemic reported in 1952, three additional epidemics occurred, including 17,786 cases in 1967, with incidence rates as high as 40.11/100,000, and 2,252 deaths, with a mortality rate of 5.08/100,000 [27]. Vaccination against JEV was launched in a few areas of Guangdong Province in 1973, and live attenuated vaccines were included in the childhood immunization program in 2004 [14]. A few studies have reported the seroprevalence of JE in Guangdong Province [28,29]. As previous studies revealed, in 2010, the positivity rate of anti-JEV IgG antibodies among the healthy population in Nanshan District, Shenzhen City, Guangdong Province, was 34.55%, among which the positivity rate among local residents was 71.13%. From 2012–2014, the positivity rate of anti-JEV IgG antibodies among the healthy population in Huaiji County, Zhaoqing City, Guangdong Province, was 85.76%, and that in Fengkai County was 62.75%. However, these studies were limited to local areas and were short-term studies. In addition, these studies were based mainly on anti-JEV IgG antibody tests. Although the level of IgG antibodies can be used as a serological marker of past exposure to JEV or successful vaccination against JE, not all IgG antibodies have neutralizing activity. In contrast, the level of neutralizing antibodies is an effective protective component against JEV and can more accurately reflect the actual immunity of the population. In particular, the eradication of JEV is almost impossible because of the persistence of infectious agents in the animal and vector life cycles, e.g., pigs and mosquitoes, respectively. Therefore, conducting postvaccination immunogenicity and durability observations for the public population is essential for formulating proper vaccination policies for JE control and prevention. In this study, we aimed to assess the degree of immunity to JE in Guangdong Province via enzyme-linked immunosorbent assay (ELISA) and microneutralization test (MNT) and to evaluate the seroprotection of the live-attenuated JE vaccine, which may guide the prevention and control of JE and the reform of vaccination programs.

## Methods

### Ethics statement

This study was approved by the Medical Research Ethics Review Committee of the Guangdong Provincial Center for Disease Control and Prevention (approval number: W96-027E-202018). For children aged 1–7 years, written informed consent was signed by their legal guardian; for minors aged 8–17 years, written informed consent was signed by both the participant and their legal guardian; and for adults aged 18 years, written informed consent was signed by the participants themselves.

### Sampling design

A cross-sectional study was performed from November 2018 to December 2022. A multistage stratified random sampling approach was used to select participants from Guangdong Province. First, three cities (Guangzhou City, Heyuan City, and Zhanjiang City) were selected on the basis of economic strata. Second, a district or county was randomly selected in each city (Panyu District in Guangzhou City, Heping County in Heyuan City, and Leizhou City in Zhanjiang City). Among them, Panyu District of Guangzhou City is an urban area, whereas Heping County of Heyuan City is a rural area, and Leizhou City of Zhanjiang City covers urban, peri-urban and rural areas. A hospital with a 2A grade or above in each district or county was subsequently chosen as a monitoring point. According to the formula:


$$\frac{Z_{\text{1}-\alpha /\text{2}}^{\text{2}}P(\text{1}-P)}{\delta^{\text{2}}}$$

With α = 0.05, δ = 0.1, and *p* = 66.47% [30], the minimum sample size for each district or county is 85. Considering nonresponse and other factors, we randomly included more than 100 samples from each hospital and investigated the basic information and immunization history. The inclusion criteria for participants were (1) had resided in the location for at least three months and (2) were in good health. The exclusion criteria for participants were (1) refusal of venous blood collection and (2) immunocompromised status or having received blood and blood products within the prior six months.

### Information collection

The basic information of the participants, including ID number, sex, age, and residential address, was collected through questionnaires. The history of participants receiving the Japanese encephalitis vaccine was sourced from the immunization program information system of the Guangdong Center for Disease Control and Prevention, which includes the vaccination date, number of doses, vaccine type, and manufacturer.

Venous blood samples (5 mL) were taken from each participant, and the serum was separated (centrifugation at 1,881 × g for 5 min) and stored below −20°C until testing.

### Anti-dengue virus IgG antibody test

ELISAs were performed on all the samples using the Panbio Dengue IgG antibody kit to detect the cross-reactivity between JEV and dengue virus. The experimental procedure was carried out strictly according to the instructions supplied with the kit. Panbio units <9 are negative, those 9–11 are ambiguous, and those >11 are positive. In accordance with the kit instructions, the specificity for dengue-negative samples was 100%, and the sensitivities for primary and secondary dengue were 87.0% and 100%, respectively.

### Anti-Japanese encephalitis virus IgG antibody (anti-JEV IgG) test

ELISAs were performed on all the samples using an InBios JE IgG antibody kit (InBios International, Inc., USA) to detect the presence of JEV-specific IgG antibodies. The experimental procedure was carried out strictly according to the instructions supplied with the kit. The immune status ratio (ISR) was calculated from the Japanese Encephalitis Recombinant Antigen (JERA) OD value/Normal Cell Antigen (NCA) OD value according to the manufacturer’s instructions. An ISR < 2.0 was considered negative, a value of 2.0–5.0 was ambiguous, and a value >5.0 was positive.

### Virus cultures and titrations

The SA 14-14-2 strain was inoculated onto a growing monolayer of BHK21 cells at a multiplicity of infection (MOI) of 0.01 and incubated at 37°C with 5% CO_2_. The virus was harvested after 75% of the cells showed a cytopathic effect (CPE). The mixture was subsequently centrifuged at 301 × g for 5 minutes and stored at −80°C until use. The virus mixture was diluted in a 10-fold gradient, inoculated into a 96-well cell plate, and incubated at 37°C with 5% CO_2_ for 5–7 days. The Reed–Muench method was used to calculate the TCID_50_ of this virus strain.

### Japanese encephalitis virus-neutralizing antibody (anti-JEV nAb) test

The anti-JEV nAb titers were detected by MNT. The serum was inactivated at 56°C for 30 min, diluted 2-fold from 1:10–1:320, and added to an equal amount of the SA 14-14-2 strain at 100 TCID_50_/50 μl. After incubation at 37°C with 5% CO_2_ for 2 hours, 100 µl of the virus–serum mixture was transferred to a monolayer of BHK21 cells and incubated at 37°C with 5% CO_2_ for 5‒7 days. The highest serum dilution wells with more than 50% cytopathic effects were recorded as the serum-neutralizing antibody titer. An antibody titer of ≥1:10 was considered positive for neutralizing antibodies [21,31].

### Statistical analysis

The data were analyzed and processed using SPSS 25 and GraphPad Prism 8. The significance level was set at 0.05. The correlations between the nAb titers and IgG antibody levels were measured using Spearman’s rank correlation coefficient. The chi-square test was used to compare the antibody positivity rates. The Mann‒Whitney U test was used to compare the antibody titers of two independent samples, and the Kruskal‒Wallis test was used to compare the antibody titers of multiple independent samples.

## Results

### Demographic information of the participants

A total of 691 participants in Guangzhou City, Heyuan City and Zhanjiang City were included from 2018–2022. Dengue IgG antibodies were detected in 50 individuals, whereas the remaining 641 were seronegative for dengue. Among the 691 participants, 356 were male (51.52%) and 335 were female (48.48%). The ages ranged from 1–99 years, with a median of 8 (interquartile range [IQR] 4–28) years. There were 113 (16.35%), 295 (42.69%) and 283 (40.96%) participants from Guangzhou City, Heyuan City and Zhanjiang City, respectively. There were 38 (5.50%), 83 (12.01%), 292 (42.26%), 29 (4.20%), and 249 (36.03%) participants from 2018–2022, respectively. In terms of vaccination doses, 305 (44.14%) participants received the JE vaccine (46 with a single dose, 257 with double doses and 2 with triple doses). Among the two participants who received triple doses, one was administered the inactivated vaccine, and the other received the live attenuated vaccine. Among them, 304 participants (44.00%) received the JE live attenuated vaccine (JEV-L), and one unique participant received the JE inactivated vaccine (JEV-I) (Table 1).

**Table 1:** Demographic information of the 691 subjects.

Group		N (%)
Sex	Male	356 (51.52)
	Female	335 (48.48)
City	Guangzhou	113 (16.35)
	Heyuan	295 (42.69)
	Zhanjiang	283 (40.96)
Age (Y)	1-2	83 (12.01)
	3-4	103 (14.91)
	5-6	109 (15.77)
	7-18	180 (26.05)
	19-39	61 (8.83)
	40-59	75 (10.85)
	≥60	80 (11.58)
Year	2018	38 (5.50)
	2019	83 (12.01)
	2020	292 (42.26)
	2021	29 (4.20)
	2022	249 (36.03)
Immunization	No^a^	386 (55.86)
	1 dose	46 (6.66)
	2 doses	257 (37.19)
	3 doses	2 (0.29)
Vaccination type	No^a^	386 (55.86)
	Live-attenuated vaccine	304 (44.00)
	Inactivated vaccine	1 (0.14)

^a^No: Including people who have not been vaccinated or those with undisclosed information.

### Determination of anti-JEV IgG antibody levels by ELISA

**Fig 1:**
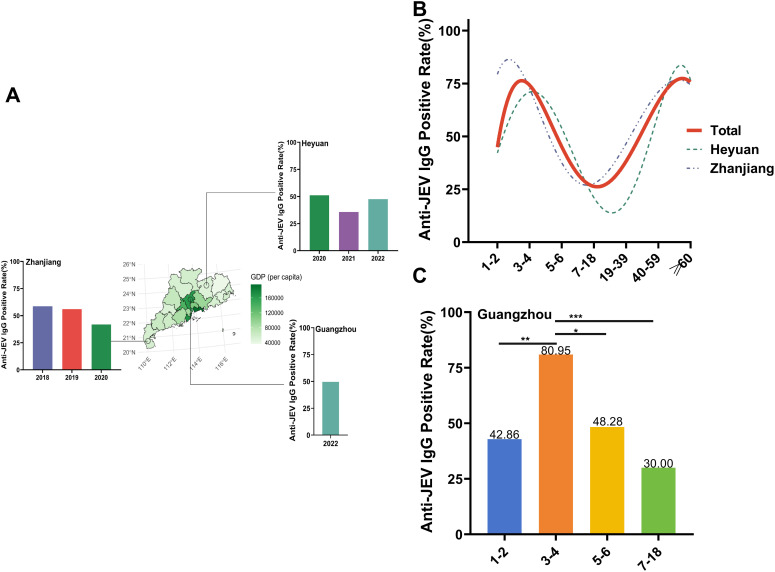
The seroprevalence of anti-JEV IgG antibodies in 641 dengue IgG antibody-negative subjects. **(A)** The background colors of the map denote the per capita GDP of all the districts in Guangdong Province, China. Bar plot illustrating anti-JEV IgG antibody-positive rates among healthy people in Guangzhou City, Zhanjiang City and Heyuan City from 2018–2022. Source of basic map: Resource and Environmental Science Data Registration and Publishing System (https://www.resdc.cn/DOI/DOI.aspx? DOIid = 121), License information: https://www.resdc.cn/NewsInfo.aspx? NewsID = 9 [32] **(B)** Trends of anti-JEV IgG antibody-positive rates by age in different regions of Guangdong Province. **(C)** Seroprevalences of anti-JEV IgG antibodies in different age groups in Guangzhou City. On the basis of the chi-square test. *p < 0.05; **p < 0.01; ***p < 0.001; ****p < 0.0001.

The anti-JEV IgG antibody positivity rate among the 691 participants was 51.37% (95% CI: 47.64–55.11%). Among the 641 dengue IgG antibody-negative individuals, the anti-JEV IgG antibody positivity rate was 48.05% (95% CI: 44.17–51.93%). The anti-JEV IgG antibody positivity rates were 49.52% in Guangzhou City, 48.04% in Heyuan City, and 47.45% in Zhanjiang City. Sex, region, year of sampling and immunization history did not significantly affect the anti-JEV IgG antibody positivity rate (all *P* > 0.05) (Table 2). The positivity rates of anti-JEV IgG antibodies among participants in Guangzhou City were 49.52% in 2022, 51.18%, 35.71%, and 47.62% in Heyuan City from 2020–2022; and 58.62%, 56.06%, and 41.88% in Zhanjiang City from 2018–2020, respectively (Fig 1A).

**Table 2:** Comparison of anti-JEV IgG antibody positivity rates in healthy people with different characteristics of 641 dengue IgG antibody-negative subjects.

Group		Test	Anti-JEV IgG positive (*n*, %)	*χ* ^2^	*P*
Sex	Male	331	151 (45.62)	1.62	0.203
	Female	310	157 (50.65)		
City^b^	Guangzhou	105	52 (49.52)	3.48	0.176
	Heyuan	191	83 (43.46)		
	Zhanjiang	163	62 (38.04)		
Age (Y)	1-2	71	32 (45.07)	100.03	<0.001
	3-4	100	73 (73.00)		
	5-6	108	52 (48.19)		
	7-18	180	40 (22.22)		
	19-39	58	24 (41.38)		
	40-59	66	43 (65.15)		
	≥60	58	44 (75.86)		
Year	2018	29	17 (58.62)	5.21	0.267
	2019	66	37 (56.06)		
	2020	287	132 (45.99)		
	2021	28	10 (35.71)		
	2022	231	112 (48.48)		
Immunization	No^a^	349	172 (49.28)	0.47	0.494
	Yes^c^	292	136 (46.57)		

^a^No: Including people who have not been vaccinated or those with undisclosed information; ^b^City: 1–18-year-old age group; ^c^Yes: Including people who received vaccination.

Overall, the anti-JEV IgG antibody positivity rates in all age groups were U shaped, with the Zhanjiang and Heyuan regions showing the same trend. The highest anti-JEV IgG antibody positivity rates were detected in the age groups of 3–4 years (73.00%) and ≥60 years (75.86%), whereas the lowest was detected in the age group of 7–18 years (22.22%). In terms of geographical distribution, the anti-JEV IgG antibody positivity rate in Heyuan City was 26.25% for the 7–18 year age group, whereas the lowest rate was in the 19–39 year age group (15%). In Zhanjiang City, the 1–2-year-old age group consisted of 5 people, with a positivity rate of 80% (Fig 1B). In Guangzhou City, the 3–4-year-old age group had the highest anti-JEV IgG antibody-positive rate (80.95%), whereas the 7–18-year-old age group had the lowest anti-JEV IgG antibody level (30%) (Fig 1C). Among the 50 dengue IgG antibody-positive participants, the anti-JEV IgG positivity rate was 94% (95% CI: 87.18–100%). Sex, region, age group, sampling year, and immunization history did not significantly affect the anti-JEV IgG antibody positivity rate (all *P* > 0.05) (S1 Table).

### Determination of anti-JEV neutralizing antibodies via MNT

**Fig 2:**
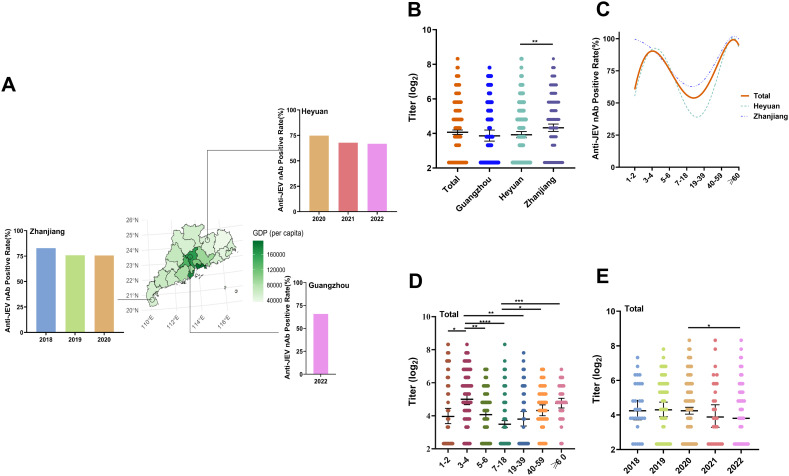
The seroprevalences and titers of anti-JEV nAb among different regions, age groups, and sampling years in Guangdong Province (based on dengue IgG antibody-negative subjects). **(A)** The background colors of the map denote the per capita GDP of all the districts in Guangdong Province, China. Bar plot illustrating anti-JEV nAb positivity rates among healthy people in Guangzhou City, Zhanjiang City and Heyuan City from 2018–2022. Source of basic map: Resource and Environmental Science Data Registration and Publishing System (https://www.resdc.cn/DOI/DOI.aspx? DOIid = 121), License information: https://www.resdc.cn/NewsInfo.aspx? NewsID = 9 [32] **(B)** Anti-JEV nAb titers against different regions of Guangdong Province. The error bars represent the geometric mean titer ± 95% confidence interval. The Mann‒Whitney U test was used. *p < 0.05; **p < 0.01; ***p < 0.001; ****p < 0.0001. **(C)** Trends of anti-JEV nAb positivity rates by age in different regions of Guangdong Province. **(D)** Anti-JEV nAb titers in different age groups in Guangdong Province. The error bars represent the geometric mean titer ± 95% confidence interval. The Kruskall-Wallis test was used. *p < 0.05; **p < 0.01; ***p < 0.001; ****p < 0.0001. **(E)** Anti-JEV nAb titers in Guangdong Province from 2018–2022. The error bars represent the geometric mean titer ± 95% confidence interval. The Kruskall-Wallis test was used. *p < 0.05; **p < 0.01; ***p < 0.001; ****p < 0.0001.

Among the 691 participants, 506 had positive anti-JEV nAb results, with a positivity rate of 73.22% (95% CI: 69.92–76.54%) and a geometric mean antibody titer (GMT) of 22.16 (95% CI: 20.25–24.08). Among them, among the 641 dengue-IgG antibody-negative participants, 462 had positive anti-JEV nAb results, with a positivity rate of 72.07% (95% CI: 68.59–74.56%) and a GMT of 21.03 (95% CI: 19.29–22.94). The positivity rates in Guangzhou City, Heyuan City, and Zhanjiang City were 65.71%, 70.46%, and 76.47%, respectively; the GMTs were 18.64, 18.77, and 25.11, respectively. The percentage of anti-JEV nAb-positive participants in Guangzhou City was 65.71% in 2022; 74.80%, 67.86% and 66.67% in Heyuan City from 2020–2022; and 82.76%, 75.76% and 75.63% in Zhanjiang City from 2018–2020, respectively (Fig 2A). The positivity rate in Zhanjiang City (76.47%) was greater than that in Heyuan City (70.46%), but the difference was not statistically significant (*χ²* = 2.47, *P* = 0.116). The GMT in Zhanjiang City (25.11) was significantly greater than that in Heyuan City (18.77) (*Z* = -3.07; *P* = 0.002) (Fig 2B).

Overall, the anti-JEV nAb positivity rates for all age groups exhibited a U-shaped trend, with the same trend in Heyuan City and Zhanjiang City. The positivity rates of anti-JEV nAb and GMT among children aged 1–2 years were 60.56% and 22.01, respectively. The positivity rates of anti-JEV nAb and GMT among children aged 3–4 years were 90.00% and 38.59, respectively, and then gradually decreased. The lowest anti-JEV nAb positivity rates (55.00%) and GMT rates (14.03) were found in the age group of 7–18 years, whereas the anti-JEV nAb positivity rate and GMT increased in people over 19 years of age, and the anti-JEV nAb positivity rate and GMT reached 94.83% and 29.24, respectively, in people over 60 years of age (Table 3 and Fig 2C and 2D).

**Table 3:** Comparison of anti-JEV nAb positivity rates and GMTs in healthy people with different characteristics among 641 dengue IgG antibody-negative subjects.

Group		Test	Anti-JEV nAb positive (*n*, %)	*χ* ^2^	*P*	GMT (95% CI)	*Z/H(K)*	*P*
Sex	Male	331	233 (70.39)	0.96	0.327	20.25 (17.88,22.94)	-1.00	0.320
	Female	310	229 (73.87)			21.86 (19.29,24.76)		
City^b^	Guangzhou	105	69 (65.71)	1.27	0.530	18.64 (14.72,23.59)	4.36	0.113
	Heyuan	191	129 (67.54)			18.64 (15.67,22.01)		
	Zhanjiang	163	117 (71.78)			23.92 (19.70,28.84)		
Age (Y)	1-2	71	43 (60.56)	73.52	<0.001	22.01 (15.45,31.12)	57.08	<0.001
	3-4	100	90 (90.00)			38.59 (31.12,47.84)		
	5-6	108	83 (76.85)			19.97 (16.56,23.92)		
	7-18	180	99 (55.00)			14.03 (11.88,16.45)		
	19-39	58	35 (60.34)			18.00 (13.00,24.93)		
	40-59	66	57 (86.36)			22.78 (18.51,28.25)		
	≥60	58	55 (94.83)			29.24 (24.42,35.02)		
Year	2018	29	24 (82.76)	7.70	0.103	22.47 (15.24,32.90)	12.28	0.015
	2019	66	50 (75.76)			24.59 (18.64,32.45)		
	2020	287	216 (75.26)			23.92 (20.82,27.28)		
	2021	28	19 (67.86)			19.03 (11.63,30.91)		
	2022	231	153 (66.23)			17.27 (14.93,19.84)		
Immunization	No^a^	349	239 (68.48)	4.92	0.027	19.56 (17.39,22.16)	-1.72	0.085
	Yes^c^	292	223 (76.37)			22.94 (20.11,26.17)		

^a^No: Including people who have not been vaccinated or those with undisclosed information; ^b^City: 1–18-year-old age group; ^c^Yes: Including people who received vaccination.

**Fig 3:**
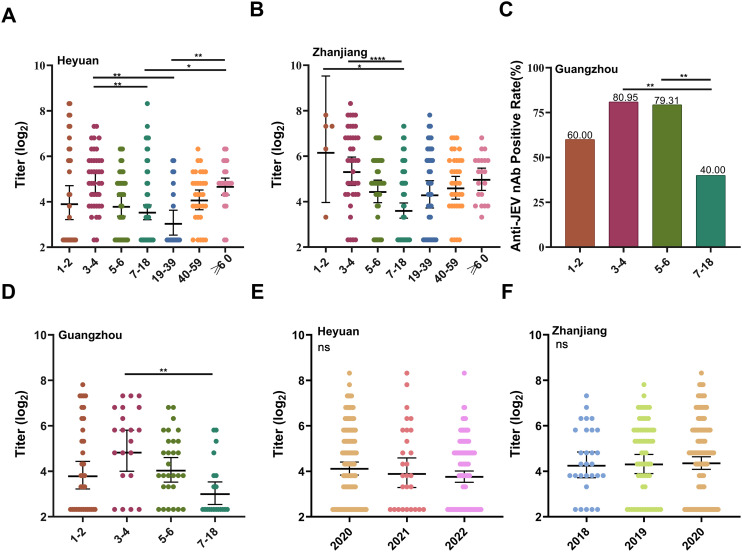
Titers of anti-JEV nAb among age groups and sampling years in different regions of Guangdong Province and anti-JEV nAb seroprevalences among age groups in Guangzhou City (based on dengue IgG antibody-negative subjects). **(A)** **(B)** Anti-JEV nAb titers among age groups in Heyuan City and Zhanjiang City. The error bars represent the geometric mean titer ± 95% confidence interval. The Kruskall-Wallis test was used. *p < 0.05; **p < 0.01; ***p < 0.001; ****p < 0.0001. **(C)** Seroprevalences of anti-JEV nAb in different age groups in Guangzhou City. On the basis of the chi-square test. **(D)** Anti-JEV nAb titers among age groups in Guangzhou City. The error bars represent the geometric mean titer ± 95% confidence interval. The Kruskall-Wallis test was used. *p < 0.05; **p < 0.01; ***p < 0.001; ****p < 0.0001. **(E) (F)** Anti-JEV nAb titers in Heyuan City and Zhanjiang City from 2018–2022. The error bars represent the geometric mean titer ± 95% confidence interval. The Kruskall-Wallis test was used. *p < 0.05; **p < 0.01; ***p < 0.001; ****p < 0.0001.

In terms of geographical distribution, the positivity rate and GMT in the 7–18-year-old age group in Heyuan City were 57.50% and 14.42, respectively, whereas the positivity rate and GMT in the 19–39-year-old age group were the lowest (35% and 9.65, respectively) (Fig 2C and 3A). In the 1–2-year-old Zhanjiang City group, five individuals had a 100% positivity rate and a GMT of 85.51 (Fig 3B). In Guangzhou City, the highest anti-JEV nAb positivity rate and GMT were found in the 3–4 year age group (80.95%, 36.00), and the lowest were found in the 7–18 year age group (40%, 9.13) (Fig 3C and 3D). The GMT was greater in 2020 (23.92) than in 2022 (17.27), and the difference was statistically significant (*P* < 0.05) (Table 3 and Fig 2E). Stratified analyses revealed that the difference in GMT between the sampling years in Heyuan City and Zhanjiang City was not statistically significant (*P* > 0.05) (Fig 3E and 3F).

Among the 50 dengue-IgG antibody-positive participants, 44 had positive anti-JEV nAb results, with a positivity rate of 88.00% (95% CI: 78.67–97.33%) and a GMT of 42.52 (95% CI: 30.91–58.49). Sex, region, age group, sampling year, and immunization history did not significantly affect antibody positivity (all P > 0.05) (S2 Table). When the participants were stratified by age group, the difference in GMT among the age groups was statistically significant (*H(K)*=16.48; *P* = 0.006); the GMT in the 1–2-year-old age group (91.77) was significantly greater than that in the ≥ 60-year-old age group (28.05). Among the 50 dengue-IgG antibody-positive participants, 13 had a clear vaccination history. The anti-JEV nAb positivity rate in vaccinated individuals (92.31%) was higher than that in unvaccinated individuals (86.49%), but the difference was not statistically significant (*χ*^*2*^ = 0.004, *P* = 0.953). The GMT in vaccinated individuals (91.14) was significantly greater than that in unvaccinated individuals (32.67), and the difference was statistically significant (*Z* = –3.042; *P* = 0.002).

**Fig 4:**
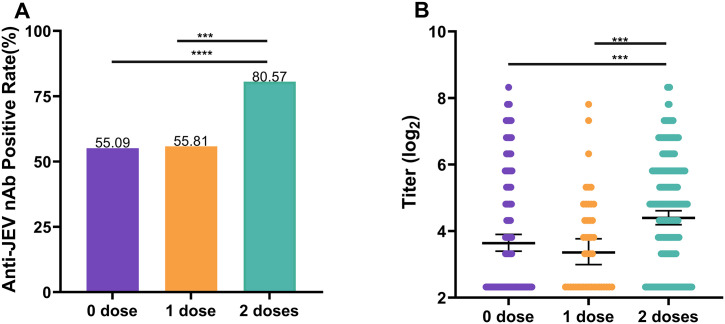
The seroprevalences and titers by vaccine dose (based on dengue IgG antibody-negative subjects). **(A)** Seroprevalences of anti-JEV nAb in participants vaccinated with different doses of JEV-L. Based on the chi-square test. *p < 0.05; **p < 0.01; ***p < 0.001; ****p < 0.0001. **(B)** Anti-JEV nAb titers in participants vaccinated with different doses of JEV-L. Error bars represent the geometric mean titer ± 95% confidence interval. The Kruskall-Wallis test was used. *p < 0.05; **p < 0.01; ***p < 0.001; ****p < 0.0001.

Among the 641 dengue-IgG antibody-negative participants, 292 had a clear vaccination history. Overall, the anti-JEV nAb positivity rate was significantly greater among vaccinated participants (76.37%) than among unvaccinated participants (68.48%) (*χ*^*2*^ = 4.92, *P* = 0.027). The GMT was greater in vaccinated participants (22.94) than in unvaccinated participants (19.56), but the difference was not significant (*Z* = -1.72; *P* = 0.085) (Table 3). Further analysis revealed that the anti-JEV nAb positivity rate and GMT were greater in those vaccinated with the live attenuated vaccine (76.63%, 23.05) than in those not vaccinated (*χ*^*2*^ = *2*2.93, *P* < 0.001; *Z* = -3.15, *P* = 0.02). In terms of the vaccine dose, the anti-JEV nAb positivity rate and GMT were greater in those who received two doses of live attenuated vaccine (80.57%, 25.92) than in those who received one dose (55.81%, 12.19) or 0 (55.09%, 16.74) (*P* < 0.001) (Fig 4A and 4B).

### Correlation and agreement between the ELISA and MNT results

**Fig 5:**
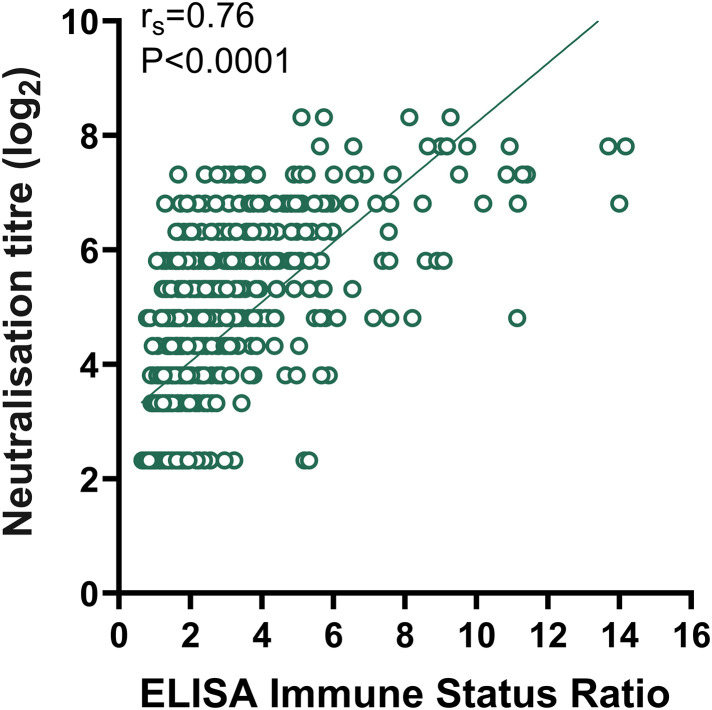
Correlations of NAb titers detected by MNT with those detected by ISR by ELISA.

Among the 691 participants, 50 dengue IgG antibody-positive participants were excluded to avoid cross-reactivity, and the remaining 641 participants were analyzed for correlation and agreement. We evaluated the correlation between immune status ratios (ELISAs) and anti-JEV nAb titers (MNTs). A positive correlation was observed between anti-JEV IgG antibody levels and anti-JEV nAb titers (*r*_*s*_ = 0.76, *P* < 0.001) (Fig 5). The results of the two assays were statistically analyzed by chi-square tests and kappa consistency tests, and the difference between the results of the two assays was statistically significant (*P* < 0.001), with a higher positivity rate with MNT detection (72.07%) than with ELISA detection (48.05%). Compared with those of the MNT, the positive percentage agreement, negative percentage agreement and agreement rate of the ELISA results were 64.50%, 94.41% and 72.85%, respectively. The kappa value of the two methods was 0.47 (Table 4). Among the participants who were vaccinated with the live attenuated vaccine, 68 were negative for anti-JEV nAbs, and 223 were positive for anti-JEV nAbs. A total of 41.70% (n = 93) of the MNT-positive samples were not detected by ELISA. Additionally, six samples tested negative for anti-JEV nAb but were judged to be positive by InBios IgG ELISA (Table 5).

**Table 4:** Agreement between the ELISA results and MNT results.

JEV IgG ELISA	MNT		
	Positive	Negative	Total
Positive	298	10	308
Negative	164	169	333
Total	462	179	641
Positive percentage agreement (%)	64.50 (95% CI:60.12 ~ 68.88)		
Negative percentage agreement (%)	94.41 (95% CI:91.02 ~ 97.81)		
Agreement rate(%)	72.85 (95% CI:69.40 ~ 76.31)		
The *p* value of Chi-square test	<0.001		
Kappa score	0.47 (95% CI:0.41 ~ 0.53)		
The *p* value of the Kappa consistency test	<0.001		

**Table 5:** Comparison of MNT results with ELISA results in participants vaccinated with live attenuated vaccines.

JEV IgG ELISA	MNT		
	Positive	Negative	Total
Positive	130	6	136
Negative	93	62	155
Total	223	68	291

## Discussion

This study analyzed the JEV serological profile of 691 participants who were recruited from Guangzhou City, Heyuan City and Zhanjiang City in Guangdong Province from 2018–2022, among whom 50 were dengue-IgG antibody-positive and 641 were dengue-IgG antibody-negative; the overall anti-JEV nAb seropositivity rate was 73.22%, with a GMT of 22.16. Among the 641 dengue-IgG antibody-negative participants, the anti-JEV nAb positivity rate determined by MNT (72.07%) was significantly higher than the anti-JEV IgG antibody positivity rate measured by ELISA (48.05%), and the two assays were positively correlated (**r_s_* *= 0.76) but showed moderate agreement (Kappa = 0.47), indicating the superior sensitivity of MNT. Age-specific seropositivity followed a U-shaped curve, peaking in the 3–4-year-old age group (90.00%) and the ≥ 60-year-old age group (94.83%) and showing the lowest values in the 7–18-year-old age group (55.00%). Vaccines are effective for preventing Japanese encephalitis, as individuals who received two doses of the live attenuated vaccine achieved the highest anti-JEV nAb positivity rate—80.57%.

In terms of methodology, ELISA and MNT showed only moderate agreement, despite a strong positive correlation between the ISR based on ELISA and the nAb titer of MNT. Moreover, 41.70% (n = 93) of the MNT-positive samples were not detected by ELISA. These results are similar to those of a randomized controlled study reported by Litzba N et al. [33]. In that study, among participants 56 days after vaccination with the Japanese encephalitis vaccine IC51 (IXIARO), 56% (n = 51) of the positive PRNT50 results were not detected by the InBios ELISA. The low antibody positivity rate detected by the InBios IgG ELISA might be due to the antigen used in the kit, which is a recombinant antigen consisting of peptide chains from different parts of the Japanese encephalitis virus antigen. The manufacturer states that the kit is not optimized for vaccine-induced seroconversion studies. Among the participants vaccinated with JEV-L in the current study, six samples tested negative for anti-JEV nAb but were positive by InBios IgG ELISA, possibly because of cross-reactivity with other flaviviruses, e.g., Zika virus, which has been imported into this area frequently within the past few decades [34–36].

The anti-JEV antibody level in healthy individuals is an important indicator of the immune status of the population and is also the most commonly used marker for disease burden estimation during surveillance. Given the above methodological comparison results, the anti-JEV IgG antibody levels detected by ELISA cannot reliably reflect the true population immunity level. In contrast, the MNT detects the level of neutralizing antibodies, an effective protective component against JEV. The total anti-JEV nAb positivity rate in Guangdong Province was 72.07%, with a GMT of 21.03, which was lower than that of the Korean population aged 15 years or older (88.02%) [37] and similar to that in Japan (68. 6%) [38], Yiwu City, Zhejiang Province (55. 83%, 13.96) [39], Chiang Mai, Thailand (52.12%) [40], and Jiangsu Province (66.47%, 12.71) [30] in China but higher than those in Alappuzha District, Kerala, India (15.91%) [41], Tongchuan City, and Shaanxi Province (24.07%, 3.04) [42] in China. Nevertheless, it was higher than the threshold of the population seropositivity rate for JE (70%) [43]. Further analysis revealed that South Korea, Japan, Yiwu City (Zhejiang Province), and Jiangsu Province presented relatively high anti-JEV nAb levels, which was attributed mainly to long-term high-coverage JE vaccination programs and a certain degree of natural infection exposure. In contrast, as low-JE-endemic regions, Alappuzha District (India) and Tongchuan City (Shaanxi Province) had limited opportunities for natural infection. Additionally, the serological survey in Alappuzha District excluded individuals who had received JE vaccination, resulting in lower overall antibody levels in these areas.

With respect to geographical and economic distribution, the less economically developed western regions of Guangdong Province, such as Zhanjiang City, were among the cities with a high incidence of JE in Guangdong Province. The aforementioned factors may likewise explain the relatively higher GMT in Zhanjiang City than in Heyuan City, as well as the relatively lower antibody-positive rate among the population aged 19–39 years in Heyuan City. In terms of sex, no bias was observed between males and females, which was consistent with the detection results for other provinces and cities [43,44].

Significant differences in anti-JEV antibody positivity rates and antibody titers were detected among age groups, indicating a U-shaped distribution trend, which is consistent with previous reports [45,46]. The anti-JEV nAb-positive rate and GMT in the 1–2-year-old age group were 60.56% and 22.01%, respectively. These data were linked to incomplete primary immunization. In the 3–4-year-old age group, the full vaccination program was completed; thus, the anti-JEV nAb-positive rate and GMT were relatively high (90.00% and 38.59, respectively), whereas among people aged 7–18 years, the anti-JEV nAb-positive rate and GMT achieved a U-shaped trend (55.00% and 14.03, respectively). The antibody levels in the 7–18-year-old age group were relatively low, which might be related to the attenuation of antibody titers over time after vaccination, relatively low vaccination coverage rates, and reduced opportunities for natural infection after the improvement of social hygiene conditions. Specifically, JEV-L was included in the immunization program in Guangdong Province in 2004. The individuals in this age group who did not complete the JEV-L were born mainly between 2000 and 2004, and they were in the stage when the JEV-L had not been fully promoted. The data from this study were strongly supported by seroepidemiological surveillance of JEV [44]. Similar serum surveys conducted in Zhejiang Province [45] and Tongchuan City [42] in China also revealed lower antibody-positive rates and GMTs in younger age groups. In contrast, the anti-JEV nAb positivity rate and GMT were relatively high in people over 40 years old. This difference might be because people in this age group have an increased chance of being bitten by mosquitoes, which results in a relatively high level of natural infection and triggers more durable and stronger anti-JEV antibodies because of the memory immune response [47,48]. Notably, among the 50 dengue IgG antibody-positive subjects, the GMT in the 1–2-year-old age group (91.77) was significantly higher than that in the ≥ 60-year-old age group (28.05). This phenomenon might be attributed to the limited sample size and the fact that the 1–2-year-old age group is in a stage of high anti-JEV nAb titer after primary immunity.

Vaccination is an effective measure against JE. A study in India revealed a significant reduction in cases in JE-vaccinated areas compared with unvaccinated areas [49]. JEV-L was shown to stimulate stronger specific immunity with higher positive serum antibody conversion rates and longer durations of antibodies [50]. According to archived data, JEV-L could achieve a 77.8–96% positivity rate for anti-JEV nAb after initial immunization and a 100% positivity rate one month after booster immunization [51]. A study in South Korea revealed that the antibody positivity rate could reach 91.1%, with a GMT of 40.9, after initial immunization in children aged 1–3 years, whereas the seropositivity rate could reach 97%, with a 6.5-fold increase in GMT after booster immunization [52]. A similar study conducted in Thailand reported that seroconversion rates were 95% at 90 days after the initial immunization and 100% at 30 days after booster immunization [53]. These data show that two doses of JEV-L are relatively more effective.

Nevertheless, our study has several limitations. First, at present, there is no laboratory testing method that can distinguish between vaccine-induced neutralizing antibodies and natural infection-induced neutralizing antibodies. However, this limitation does not affect our conclusions, as there have been fewer confirmed cases of JE in Guangdong Province in recent years [27,54], a relatively low positivity rate of mosquitoes carrying JEV in mosquito-borne monitoring [55,56], and a relative decline in the rate of natural infection. Second, we used the attenuated JEV strain in this study for measuring neutralizing antibodies, the epitopes of which may differ from those of the currently circulating wild-type strains. Third, we were unable to track the kinetic attenuation of anti-JEV nAbs in each subject. In the future, tracking at multiple time points is needed to further reveal the long-term persistence trend of anti-JEV nAbs, which will help us fully understand the immune persistence of the vaccine. Finally, the study covered only three cities in Guangdong Province. Moreover, the age structure in the actual sampling of these three cities was unbalanced—the subjects in Guangzhou City were concentrated in the 1–18-year-old age group, while those in Heyuan City and Zhanjiang City covered all the age groups. To address this imbalance, in our analysis, we first compared the three cities among the 1–18-year-old age group and then conducted separate stratified evaluations on the full-age data from Heyuan City and Zhanjiang City. In the future, the sample size should be further expanded, and larger-scale serological monitoring with unified age-stratified sampling should be conducted at more research sites to increase the representativeness of the data.

## Conclusion

In conclusion, we determined the immune dynamics of antibodies against JE in healthy individuals in Guangdong Province from 2018–2022. These data support the formation of an initial immune barrier. However, the antibody positivity rate was relatively low in the 7–39-year-old age group. A strongly recommended extra booster vaccination is essential for adult individuals in Guangdong Province, as well as in the remaining provinces in China and even in countries in Southeast Asia, which still currently suffer from JEV endemicity. In addition, JEV-I should also be considered for booster vaccination in both children and adults. Nevertheless, evidence-based booster studies need to be considered for either JEV-L or JEV-I vaccines.

## Supporting information

S1 Table Comparison of anti-JEV IgG antibody-positive rates in healthy people with different characteristics of 50 dengue IgG antibody-positive subjects. ^a^No: Including people who have not been vaccinated or those with undisclosed information; ^b^City: 1–18-year-old age group; ^c^Yes: Including people who received vaccination.
(DOCX)

S2 Table Comparison of anti-JEV nAb-positive rates and GMTs in healthy people with different characteristics of 50 dengue IgG antibody-positive subjects. ^a^No: Including people who have not been vaccinated or those with undisclosed information; ^b^City: 1–18-year-old age group; ^c^Yes: Including people who received vaccination.
(DOCX)

S1 Data The data used in this study. (XLSX)
