# Supplementary material for: Unveiling immunity dynamics: Serological characteristics of antibodies against Japanese encephalitis virus in Guangdong, China
Source: PLoS Negl Trop Dis. 2025 Oct 22;19(10):e0013629. doi: 10.1371/journal.pntd.0013629 (PMC12543178; doi:10.1371/journal.pntd.0013629)
Supplement: S1 Table — aNo: Including people who have not been vaccinated or those with undisclosed information; bCity: 1–18-year-old age group; cYes: Including people who received vaccination. (DOCX) [file pntd.0013629.s001.docx]

**Supplemental Table 1. Comparison of anti-JEV IgG antibody-positive rates in healthy people with different characteristics of 50 dengue IgG antibody-positive subjects**

| Group | | Test | Anti-JEV IgG positive (*n*, %) | *χ*^2^ | *P* |
| --- | --- | --- | --- | --- | --- |
| Sex | Male | 25 | 24(96.00) | 0.00 | >0.999 |
|  | Female | 25 | 23(92.00) |  |  |
| City^b^ | Guangzhou | 8 | 7(87.50) | 1.59 | >0.999 |
|  | Heyuan | 6 | 6(100.00) |  |  |
|  | Zhanjiang | 2 | 2(100.00) |  |  |
| Age(Y) | 1-2 | 12 | 11(91.67) | 3.04 | >0.999 |
|  | 3-4 | 3 | 3(100.00) |  |  |
|  | 5-6 | 1 | 1(100.00) |  |  |
|  | 7-18 | 0 | - |  |  |
|  | 19-39 | 3 | 3(100.00) |  |  |
|  | 40-59 | 9 | 9(100.00) |  |  |
|  | ≥60 | 22 | 20(90.91) |  |  |
| Year | 2018 | 9 | 9(100.00) | 2.69 | 0.727 |
|  | 2019 | 17 | 15(88.24) |  |  |
|  | 2020 | 5 | 5(100.00) |  |  |
|  | 2021 | 1 | 1(100.00) |  |  |
|  | 2022 | 18 | 17(94.44) |  |  |
| Immunization | No^a^ | 37 | 35(94.59) | - | >0.999 |
|  | Yes^c^ | 13 | 12(92.31) |  |  |

^a^No: Including people who have not been vaccinated or those with undisclosed information; ^b^City:1–18-year-old age group; ^c^Yes: Including people who received vaccination
