# Supplementary material for: Unveiling immunity dynamics: Serological characteristics of antibodies against Japanese encephalitis virus in Guangdong, China
Source: PLoS Negl Trop Dis. 2025 Oct 22;19(10):e0013629. doi: 10.1371/journal.pntd.0013629 (PMC12543178; doi:10.1371/journal.pntd.0013629)
Supplement: S2 Table — aNo: Including people who have not been vaccinated or those with undisclosed information; bCity: 1–18-year-old age group; cYes: Including people who received vaccination. (DOCX) [file pntd.0013629.s002.docx]

**Supplemental Table 2. Comparison of anti-JEV nAb-positive rates and GMTs in healthy people with different characteristics of 50 dengue IgG antibody-positive subjects**

| Group | | Test | Anti-JEV nAb positive(*n*, %) | *χ*^2^ | *P* | GMT  (95% CI) | *Z/H(K)* | *P* |
| --- | --- | --- | --- | --- | --- | --- | --- | --- |
| Sex | Male | 25 | 22(88.00) | 0.00 | >0.999 | 53.08  (32.45,87.43) | -1.62 | 0.105 |
|  | Female | 25 | 22(88.00) |  |  | 34.06  (22.47,51.98) |  |  |
| City^b^ | Guangzhou | 8 | 7(87.50) | 1.59 | >0.999 | 70.03  (22.01,222.86) | 1.20 | 0.587 |
|  | Heyuan | 6 | 6(100.00) |  |  | 134.36  (73.52,243.88) |  |  |
|  | Zhanjiang | 2 | 2(100.00) |  |  | 79.34  (0.97,6472.02) |  |  |
| Age(Y) | 1-2 | 12 | 11(91.67) | 2.34 | 0.893 | 91.77  (45.25,186.11) | 16.48 | 0.006 |
|  | 3-4 | 3 | 3(100.00) |  |  | 100.43  (4.82,2076.59) |  |  |
|  | 5-6 | 1 | 1(100.00) |  |  | 56.00  (-) |  |  |
|  | 7-18 | 0 | - |  |  | - |  |  |
|  | 19-39 | 3 | 3(100.00) |  |  | 89.26  (14.93,537.45) |  |  |
|  | 40-59 | 9 | 7(77.78) |  |  | 24.08  (10.63,54.57) |  |  |
|  | ≥60 | 22 | 19(86.36) |  |  | 28.05  (18.77,41.93) |  |  |
| Year | 2018 | 9 | 7(77.78) | 2.13 | 0.819 | 26.17  (11.16,60.97) | 8.25 | 0.083 |
|  | 2019 | 17 | 15(88.24) |  |  | 31.12  (19.29,50.21) |  |  |
|  | 2020 | 5 | 5(100.00) |  |  | 91.14  (51.27,163.14) |  |  |
|  | 2021 | 1 | 1(100.00) |  |  | 112.00  (-) |  |  |
|  | 2022 | 18 | 16(88.89) |  |  | 56.10  (29.65,106.89) |  |  |
| Immunization | No^a^ | 37 | 32(86.49) | 0.00 | 0.953 | 32.67  (23.43,45.25) | -3.04 | 0.002 |
|  | Yes^c^ | 13 | 12(92.31) |  |  | 91.14  (44.94,184.82) |  |  |

^a^No: Including people who have not been vaccinated or those with undisclosed information; ^b^City:1–18-year-old age group; ^c^Yes: Including people who received vaccination
